# The effect of transcutaneous electrical nerve stimulation (TENS) on pain and vital signs during chest tube removal

**DOI:** 10.12669/pjms.42.2.12653

**Published:** 2026-02

**Authors:** Acelya Turkmen, Alper Avci, Ilknur Tura, Sevilay Erden

**Affiliations:** 1 Acelya Turkmen Department of Nursing, Faculty of Health Sciences, Cukurova University, Adana, Turkiye; 2 Alper Avci Department of Thoracic Surgery, Faculty of Medicine, Cukurova University, Adana, Turkiye; 3 Ilknur Tura Department of Nursing, Faculty of Health Sciences, Cukurova University, Adana, Turkiye; 4 Sevilay Erden Department of Nursing, Faculty of Health Sciences, Cukurova University, Adana, Turkiye

**Keywords:** Chest tube removal, Pain, Postoperative, Pain management, Transcutaneous electric nerve stimulation, Vital Signs

## Abstract

**Objective:**

The aim of the study was to determine the effects of transcutaneous electrical nerve stimulation (TENS) on pain and vital signs during chest tube removal (CTR).

**Methodology:**

This randomized controlled study was conducted with a group of patients treated in the thoracic surgery unit of a university hospital between May 2023 to September 2023. Participants were equally allocated using simple randomization. This randomized-controlled study collected data 60 thoracic surgery patients using Patient Information Form (PIF) and the Numerical Rating Scale (NRS). TENS was applied for a total of one hour, 2-3 cm from the chest tube site. The pain level and vital signs of the patients were assessed at immediately before (T1), during (T2) and immediately after (T3) the CTR in all groups.

**Results:**

Pain levels were significantly lower in the TENS group compared to the control group (p < 0.05). In addition, a significance was found between the groups at T2 for Heart Rate (HR), T2-T3 for Systolic Blood Pressure (SBP) and T2-T3 for Respiratory Rate (RR). HR, SBP and RR were statistically significant at T2 time (p<0.05) in this group.

**Conclusion:**

TENS effectively reduces acute procedural pain and provide maintain stable vital signs as a safe complementary method in multimodal pain management.

## INTRODUCTION

Chest tube placement is routinely performed after thoracic surgery to drain the pleural cavity and support respiratory and hemodynamic functions.^1^ The chest tube is removed within 24-48 hours, unless there are any complications after the surgery.^2^ During the chest tube removal (CTR), the patients experience pain due to the separation of adhesion and the physical impact on the surrounding tissues.^1^,^2^ CTR, may be associated with a severe transient pain who have undergone surgery. Due to the abundance of sensory fibers in the pleura of the lung, the pain associated with CTR may be related to burning, pressure and tightness. This condition may cause fear and anxiety in patients.^2^ Reducing the pain caused by the chest tube is important for effective breathing. Uncontrolled pain can cause negative outcomes such as forced inspiration/expiration, the difficulty in coughing and accumulation of secretions, atelectasis, hypoxemia, infection and accordingly, deterioration in hemodynamic parameters.^3^

There are many different pharmacological and non-pharmacological pain management methods^2^ and pharmacological methods (such as opioid and non-steroidal inti-inflammatory analgesics) are generally used to control acute pain during CTR.^2^,^4^ However, pain guidelines and studies recommend multimodal analgesia to restrict the use of opioids that negatively affect respiratory function. In this context, analgesics should be used together with non-pharmacological methods and appropriate nursing care in order to reduce the level of pain and provide an adequate analgesic effect when CTR.^5^ In the literature non-pharmacological methods such as cold application, massage acupressure and transcutaneous electrical nerve stimulation (TENS) have been used in pain management.^2^ One of the non-pharmacological methods, TENS is used in pain management in thoracic surgery.^2^ In this method, the frequency and intensity of electrical stimulation can be adjusted according to the analgesic needs of patients.^6^

Several studies have reported that TENS reduces pain and positively affects patients’ hemodynamic parameters, such as respiratory function.^3^,^7^ Research has shown that TENS is effective in reducing procedural pain following thoracotomy.^3^,^8^ There are studies on the use of TENS to reduce pain caused by CTR after different surgical interventions.^2^,^8^ Santuzzi et al., stated that TENS reduces systemic blood pressure and pulse by blocking the sympathetic flow to the heart and vessels.^9^ This study contributes to the literature by addressing the limited evidence on the non-pharmacological management and nursing care of acute procedural pain during CTR. While pharmacological interventions are frequently used, their side effects and limitations necessitate the exploration of complementary approaches. TENS, as a non-invasive and cost-effective method, has been investigated in various clinical contexts; however, its effectiveness and integration into nursing care during CTR remains understudied. This research provides valuable evidence through a randomized controlled design, demonstrating the impact of TENS not only on pain reduction but also on vital signs stabilization during the procedure. In this regard, this study was conducted to overcome this limitation by examining the effect of TENS after thoracic surgery on pain and vital signs during CTR.

## METHODOLOGY

This study adopted a randomized controlled experimental design to determine the effect TENS on pain and vital signs during CTR. The study was conducted with a group of patients treated in the thoracic surgery unit of a university hospital between May 2023 to September 2023. The thoracic surgery unit has 16 beds and approximately 70% of patients have a chest tube following surgery. In the thoracic surgery unit, pain assessment is not performed on patients during CTR and routine analgesics are not administered before the procedure. The sample of the study consisted of patients who were hospitalized in the thoracic surgery clinic of a university hospital between May and September 2023 and met the following inclusion criteria.

### Ethical approval:

**Fig.1:**
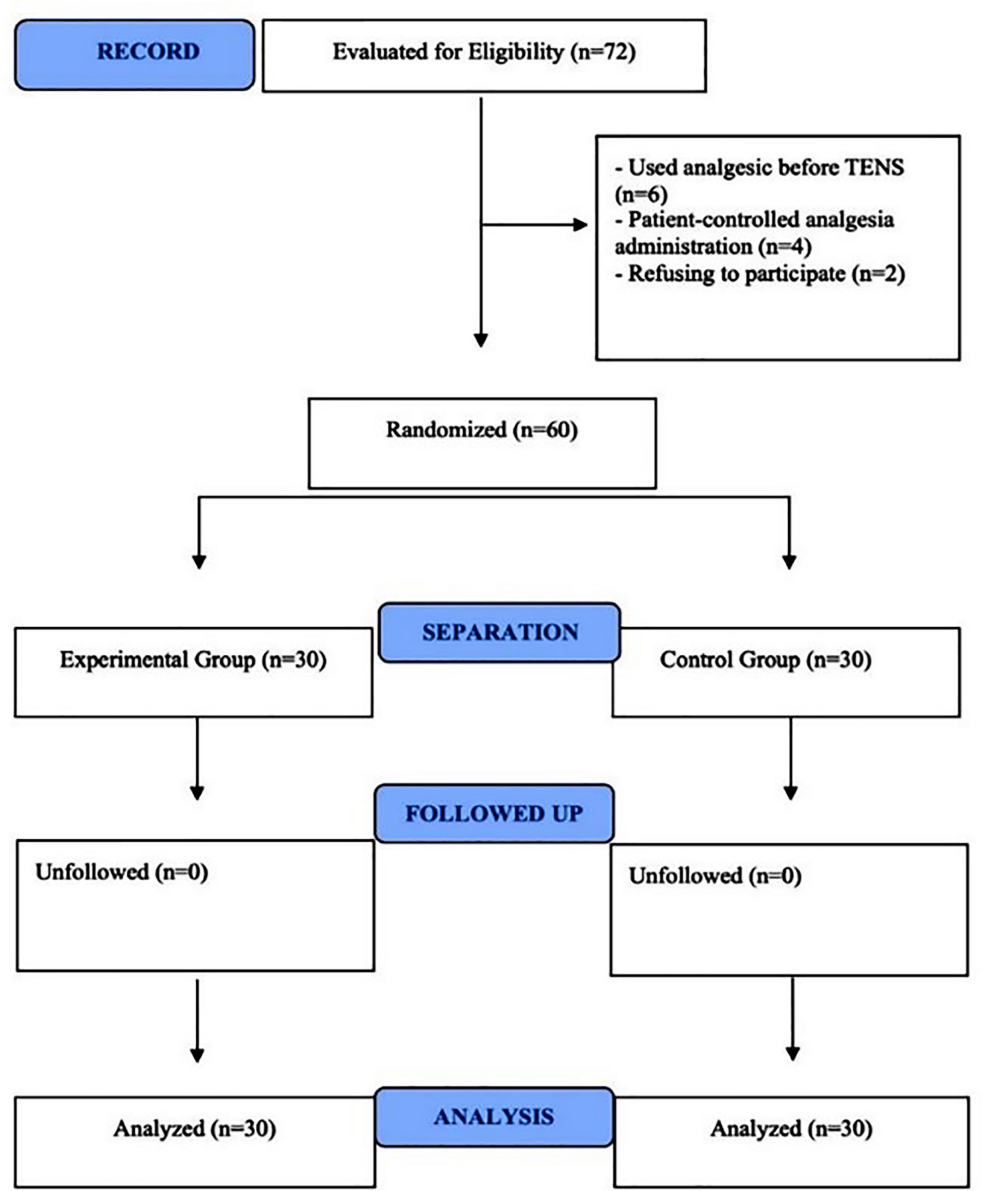
Consort 2010 Flow Diagram.

Research ethics approval was granted by the Cukurova University Non-Interventional Clinical Research Ethics Committee Institutional Review Board (May 5, 2023, 133 approval number) and informed consent were obtained in accordance with the Declaration of Helsinki. The study was registered on ClinicalTrials.gov (NCT05927168) and reported following CONSORT guidelines (Fig.1).

### Inclusion criteria:

Age ≥18 years; verbal communication ability; presence of a chest tube due to lung cancer; absence of pacemaker, arrhythmia, chronic pain, psychiatric disorders, substance abuse, or prior TENS/opioid use; no postoperative epidural analgesia; being conscious and oriented; and no contraindications to TENS application.

### Exclusion Criteria:

Seventy two patients were included in the study group. However, 12 patients were excluded: six who had used analgesics before the TENS application, two who did not agree to participate in the study and four who had used patient controlled analgesia. The sample size was calculated using G*Power v3.1.9.7 based on the difference between two independent means. Referring to a similar study,^10^ a total of 60 patients was determined to achieve 80% power, a 95% confidence interval, a significance level of 0.05 and an effect size (Cohen’s value) of 0.84.

### Measurements:

Data were collected using the Patient Information Form (PIF), developed by the researchers based on relevant literature,^3^,^8^-^11^ and the Numerical Rating Scale (NRS). The PIF included demographic and clinical variables such as age, gender, education, diagnosis, surgical details, pain perception and vital signs. The NRS assessed pain intensity on a 0-10 scale, where 0 = no pain, 1-3 = mild, 4-6 = moderate, 7-9 = severe and 10 = unbearable pain.

### TENS device:

A portable, battery-operated, dual-channel TENS device with adjustable settings was used. Conventional TENS was selected for its safety in acute pain, delivering high-frequency (50-100 Hz), short pulse duration (200 µs) and low-amplitude (1-100 mA) stimulation to induce a tingling sensation without muscle contraction.

### Procedure:

Written and verbal informed consent was obtained from all participants. Patients were randomly assigned to experimental and control groups. Descriptive data collection, TENS application and pain assessment were conducted by the researcher. In the experimental group, TENS application was started 45 minutes before the CTR. When the chest tube was removed, the electrodes were left on the patient’s body and only the current cables were removed. After the procedure, TENS was applied for 15 minutes, for a total of one hour. The pain level of the patients was assessed with NRS at T1, T2 and T3 times in the experimental and the control group. The patients were monitored and their vital signs were recorded immediately before TENS (T1), during TENS (T2) and after TENS (T3) the CTR.

### Implementation of the TENS:

**Fig.2:**
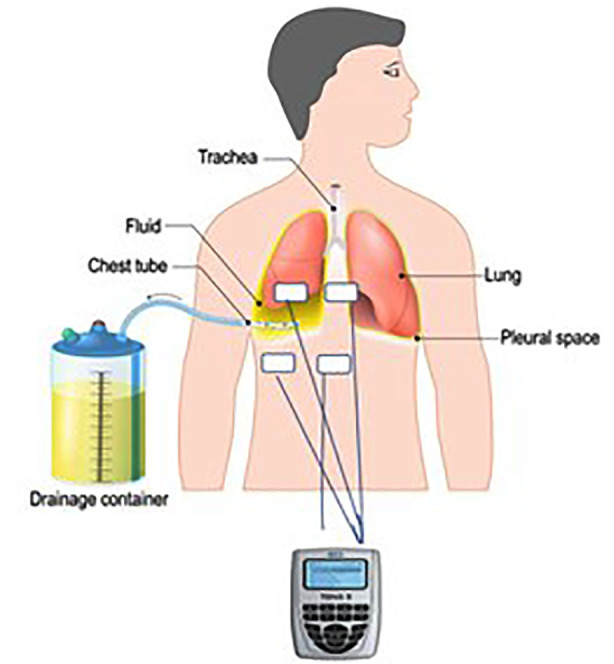
TENS electrode placement diagram for a patient with chest tubes.

In the experimental group, TENS was applied using two electrodes placed 2-3 cm above and two electrodes placed 2cm below the chest tube (Fig.2). The conventional parameters were used (frequency: 50-100 Hz; pulse width: 200 μs; duration: 1 hour). The amplitude was adjusted based on patient feedback to produce a non-disturbing tingling sensation. After the session, the electrodes were removed and cleaned with gauze.

### Randomization and Blinding:

Participants were equally allocated using simple randomization by an independent nurse via SAS software. Due to the nature of TENS, blinding of patients was not feasible. Data were collected by two independent nurse researchers, then groups were coded as 1 and 2 and the data were digitally archived.

### Statistical analysis:

In statistical analysis, number, percentage, mean, minimum, maximum, and standard deviation were calculated. The Chi-Square Test (χ^2^) test was used to analyze the descriptive characteristics of the patients and to compare the categorical variables in the independent groups. Also, the independent-t test was used to compare the TENS-control groups with the times; in repeated measurements, the analysis of variance was used. As a result of the analysis, the data that gave rise to the significance was evaluated using the Bonferroni correction, one of the post hoc analysis tests. *p=0.05* was considered statistically significant in all tests used.

## RESULTS

The distribution of the descriptive characteristics of the patients is given in Table-I. There were no significant differences between the two groups in terms of age, BMI, incision size, duration of surgery, postoperative days, gender distribution, presence of chronic disease, type of surgery, resection type and chest tube side. Accordingly, the TENS and control groups are homogeneous in terms of demographic characteristics (p> .05).

**Table-I:** Sample characteristics, overall and by group (n=60).

Descriptive Characteristics		TENS Group Mean±SD	Control Group Mean±SD	p/t
Age (years)		51.00±18.97	49.53±17.98	t=0.307 p=.760
BMI (kg/m^2^)		25.27±5.26	24.85±3.60	t=0.362 p=.719
Incision size (cm)		7.63±5.63	8.93±6.69	t=0.814 p=.419
Duration of surgery (min)		133.90±51.08	122.83±45.85	t=0.883 p=.381
Postoperative days		3.00±1.61	2.97±1.82	t=0.75 p=.883
		(n=30), n (%)	(n=30) n (%)	p/x^2^
Gender	Male	22 (73.3)	20 (66.7)	x^2^=0.317 p=.779
	Female	8(26.7)	10 (33.3)	
Chronic disease*	Yes	21 (70)	22 (73.3)	x^2^=0.082 p=.774
	No	9 (30)	8 (26.7)	
Type of Surgery	VATS	21 (70.0)	19 (63.3)	x^2^=0.320 p= .584
	Thoracotomy	9 (30.0)	11 (36.7)	
Based on resection	Lymph node sampling	4 (13.3)	6 (20.0)	x^2^=2.998 p=.392
	Wedge resection	12 (40.0)	13 (43.3)	
	Lobectomy	6 (20.0)	8 (26.7)	
	Tumor excision	8 (26.7)	3 (10.0)	
Chest tube side	Right	22 (73.3)	20 (66.7)	x^2^=0.317 p=.573
	Left	8 (26.7)	10 (33.3)	

*χ*^2^: Chi-square test; t: İndependent Simple t Test; BMI: Body Mass Index; VATS: Video-assisted thoracoscopic surgery, Chronic disease*; HD: Heart Disease, HT: Hypertension; DM: Diabetes Mellitus

The effect of TENS on pain levesl is shown in Table-II. The mean pain levels were compared at the three time points (T1, T2 and T3) and found to be statistically significant (*p<.001*). The mean pain levels at T1 were 2.30 ±1.643 in the TENS group and 4.63 ±2.399 in the control group. At T2, the mean pain levels were 4.29 ±2.235 in the TENS group and 8.33 ±1.918 in the control group. At T3, the mean pain levels were 0.73 ±0.944 in the TENS group and 5.63 ±2.189 in the control group. Bonferonni test, which is one of the post-hoc tests, was used to determine intra-group significance in both the experimental and control groups, and it was determined that this difference was due to T1 and T3 times.

**Table-II:** Pain levels of the groups according to the times (n=60).

Pain levels	Times/Mean±SD			
	T1	T2	T3	χ^2^/p
TENS Group	2.30±1.643	4.29±2.235	0.73±0.944	χ^2^=8.995 *p<.001*
Control Group	4.63±2.399	8.33±1.918	5.63±2.189	
*t/p*	p<.001*	p<.001*	p<.001*	

t:Independent Sample t Test;^2^: Friedman Test; SD: Standart Deviation; T1=Pre-procedure; T2= During the procedure; T3= Post procedure

The distribution of vital signs of the groups according to time is shown n Table-III. A significance was found between the groups at T2 for HR, T2-T3 for SBP and T2-T3 for RR (*p<.05*). In the group receiving TENS at T2, heart rate (81.53±15.005), systolic blood pressure (117.27 ±16.719) and respiratory rate (20.87±4.024) were found to be significantly lower than in the control group. The Bonferroni test was used to determine intra-group significance in both the experimental and control groups, and it was found that the difference was due to T2.

**Table-III:** Vital signs of the groups according to times (n=60).

Vital Signs T1		Times/ Mean±SD			
		T2	T3	χ^2^/p
HR	TENS Group	81.70±17.43	81.53±15.00	81.20±16.50	χ^2^=9.144 p<.001
	Control Group	86.83±12.57	99.97±13.74	86.83±12.17	
*t* *p*		-1.3 .195	-4.731 <.001*	-1.310 .195	
SBP	TENS Group	122.47±15.65	117.27 ±16.71	121.37±13.66	χ^2^=11.242 p<.001
	Control Group	119.33±11.63	131.43±16.67	132.13±14.90	
*t* *p*		0.880 .383	-3.286 .002	-2.643 .005	
DBP	TENS Group	76.00±8.02	76.83±8.04	81.40±9.18	χ^2^=5.990 p=004
	Control Group	76.10±6.82	80.83±9.69	83.60±8.76	
*t* *p*		-0.552 .959	-1.740 .087	-0.949 .346	
BT	TENS Group	36.46±0.36	36.47±0.18	36.37±0.36	χ^2^=0.500 p=.609
	Control Group	36.32±0.34	36.39±0.24	36.27±0.34	
*t* *p*		1.877 .066	1.557 .098	1.445 .154	
RR	TENS Group	21.60±1.99	20.87±4.02	22.17±1.99	χ^2^=3.671 p=.032
	Control Group	22.40±1.85	23.93±3.03	22.40±1.85	
*t* *p*		-1.612 .112	-3.331 .002	-3.319 .002	
SPO2	TENS Group	96.23±1.79	95.77±1.88	96.47±1.83	χ^2^=1.150 p=.324
	Control Group	96.67±1.78	96.50±1.25	96.73±1.57	
*t* *p*		-0.216 .830	-1.773 .082	-0.604 .548	

t: Independent Sample t Test; χ^2^: Friedman Test; SD: Standart Deviation; HR: Heart Rate; SBP: Systolic Blood Pressure; DBP: Diastolic Blood Pressure; BT: Body temperature; RR: Respiratory Rate; SPO2:Oxygen saturation; T1=Pre-procedure; T2=During the procedure; T3= Post procedure.

## DISCUSSION

In this study, it was determined that the mean pain scores were lower in the group that received TENS immediately before, during, and after CTR. In addition, it was found that the vital signs of the TENS group were within normal limits compared to the control group.

There was a difference between the pain levels of the patients in the TENS and control groups at all times. Furthermore, mean pain levels were found to be lower in the TENS group than in the control group at all times (pre, during and post procedure). This finding is supported by the findings of similar studies in the literature. Cardinali et al. implemented TENS before and 30 minutes after the chest tube was removed and the mean pain of the experimental group was found to significantly decrease.^8^ In a meta-analysis study on patients who underwent pulmonary surgery, Zhou et al. found that TENS was an effective method in postoperative pain management.^6^ The results demonstrate the significance of nurses’ active participation in the evaluation and management of procedural pain.

Husch et al., evaluated the effectiveness of TENS after thoracotomy and found that it significantly reduced the level of pain following the procedure. In a study with patients who had undergone coronary bypass grafting,^12^ Hatefi et al. found that the pain level during CTR was lower in the TENS group.^2^ Similarly, some other studies have indicated that TENS application reduces the level of procedural pain following surgery.^7^,^11^-^13^ These findings demonstrate not only the potential of TENS in reducing acute pain but also its value as a safe and effective complementary technique for managing procedural pain associated with invasive interventions. Therefore, integrating TENS into analgesia protocols during procedures such as CTR following thoracic surgery may enhance multidisciplinary pain management methods.^2^-^4^ Integration of TENS into postoperative care aligns with evidence-based nursing practices that aim to promote patient comfort and support faster recovery.

In addition to pain management, vital signs were also evaluated immediately before, during and after the procedure in this study. It was determined that the systolic/diastolic blood pressure and pulse and respiratory rate of the TENS group within the normal range as opposed to the control group, which showed significantly higher values. This finding is well-supported in the literature. Jin et al. found that the cardiopulmonary parameters of patients treated with TENS decreased.^10^,^11^ In a Cochrane review study, the respiratory rate, systolic blood pressure and heart rate of the TENS-administered group were lower.^14^ Monitoring and interpreting changes in vital signs during CTR is an essential component of clinical assessment. The use of TENS in nursing practice aids pain control and contributes to regulating vital parameters by reducing the sympathetic activation associated with pain and anxiety.

In a meta-analysis study, it was stated that TENS improved lung functions.^6^ Some other studies have also shown that the vital signs (SBP/DBP, HR and RR) of patients who have undergone a procedural intervention are within normal limits.^10^,^15^-^17^ Kumar showed that procedures such as CTR cause pain and thus activate the neuroendocrine system and cause deterioration in hemodynamic parameters (pulse, BP, RR).^18^ Considering all these, pain during procedural interventions should be controlled and vital signs should be kept within normal limits since hemodynamic changes after thoracic surgery may cause respiratory complications, such as decreased secretion, atelectasis and hypoxia in patients, as well as negative outcomes such as delayed recovery and prolonged hospital stay.^10^,^15^-^19^

### Limitations:

Firstly, the study was conducted in a single hospital and clinic is a limitation. In addition, pain perception is a subjective experience that can be influenced by individual factors such as psychological state, cultural background and previous pain experiences and these factors could not be fully controlled in this study. Secondly, follow-up was limited to the period immediately before, during and after chest tube removal; therefore, the long-term effects of TENS on postoperative pain and recovery could not be assessed. Finally, vital signs were used as indirect indicators of physiological response; more objective measurements, such as stress hormone levels or advanced cardiopulmonary monitoring, could provide a more comprehensive understanding of the physiological effects of TENS.

## CONCLUSIONS

As a result, this study contributes to the literature by addressing the limited evidence on the non-pharmacological management of acute procedural pain during chest tube removal (CTR). While pharmacological interventions are frequently used, their side effects and limitations necessitate the exploration of complementary approaches. TENS, as a non-invasive and cost-effective method, has been investigated in various clinical contexts; however, its effectiveness during CTR remains understudied. This research provides valuable evidence through a randomized controlled design, demonstrating the impact of TENS not only on pain reduction but also on vital signs stabilization during the procedure. By highlighting the physiological and analgesic benefits of TENS in this specific context, the study supports its integration into clinical protocols for pain management in thoracic care; however, further large-scale, multicentre studies are needed before it can be implemented more widely. Furthermore, it encourages interdisciplinary collaboration and the advancement of multimodal pain control strategies in different surgical procedures in the future research.

### Authors Contribution:

**AT:** Conceived, designed and did statistical analysis & editing of manuscript, is responsible for integrity of research.

**AT, AA, IT and SE:** Did data collection and manuscript writing. Critical Review.

**AT:** Critical review and final approval of manuscript.
